# Energy drink consumption in young subjects: a growing problem

**DOI:** 10.11604/pamj.2022.43.107.36067

**Published:** 2022-10-27

**Authors:** Anna Vittoria Mattioli, Antonio Manenti, Alberto Farinetti

**Affiliations:** 1 Surgical, Medical and Dental Department of Morphological Sciences Related to Transplant, Oncology and Regenerative Medicine, University of Modena and Reggio Emilia, Modena, Italy,; 2 Department of Medical and Surgical Sciences for Children and Adults, University of Modena and Reggio Emilia, Modena, Italy

**Keywords:** Energy drinks, caffeine, young, health

## To the editors of the Pan African Medical Journal

We have read with great interest the paper “Caffeinated beverage consumption among adolescents in Sagamu, Nigeria: implications for health promotion” by Sholeye O *et al*. [1] and we found it of importance with a view to health prevention in young subjects. This cross-sectional study carried out among 350 adolescents evaluated the pattern of caffeinated drink consumption among in-school adolescents in Sagamu, Ogun State, Nigeria. Authors concluded that consumption of caffeinated beverages was high among adolescents in Sagamu.

We find the findings reported in the original article of great interest and would like to provide contribute to the discussion. The manuscript highlights some important aspects for the health of young people. The first aspect is the growing diffusion of the use of energy drinks in the world. This data emerges from recent literature and is constant [2]. To understand the phenomenon of the increasing consumption of energy drinks among adolescents, it is important to identify the motivations and triggers behind it. Energy drinks (EDs) are widespread among young people who consume them for different reasons: the main reason is to increase concentration and attention during intense study sessions or before sporting activity, however, in some cases they are taken during recreational activities, in some cases mixed with alcohol which enhances its effects [2,3]. One of the reasons leading to increased consumption is poor knowledge of the effects of EDs on health and of the ingredients included in these beverages.

The main ingredient is caffeine, however, young people think that the amount of caffeine is similar to that contained in sports drinks and soft drinks. Additionally, weekly consumption of EDs is high due to teens' misperception that they are safe drinks [4]. These aspects indicate the need for educational interventions aimed at developing adolescents' knowledge and skills related to energy drinks and their effects on health. Another interesting aspect reported by the authors [1] is that a large number of respondents reported cravings for caffeinated beverages. Food craving has been reported in several manuscript as a way to cope with stress during the last 2 years [5]. The craving for ED could also be induced by the boredom and social isolation that has been induced in an attempt to reduce infections from SARS-CoV-2.

During the recent quarantine induced by the spread of COVID-19, changes in drinking habits have been reported that have also affected young subjects [6]. We recently analyzed on a group of Italian students´ changes in ED´s consumption habits occurred during the social quarantine for COVID-19. In our experience the frequency and amount of energy drink consumption was increased mainly in male. Students also reported a change in the motivation for taking EDs. Before quarantine, the main reason for drinking was “to increase concentration during exam preparation”, while during quarantine the main reason was “to increase attention by playing video games”. In addition, the group that increased ED consumption also had a reduction in sleep hours. The effects on sleep are partly attributable to the increase in time spent using tablets and electronic devices at night. It is interesting to observe that the changes induced by COVID-19 on young people have mainly affected recreational drinks.

Sholeye *et al*. also reported that over 10% had to increase the number of drinks to obtain the desired excitement. Caffeine is known to be addictive and regular coffee users report withdrawal symptoms [7]. A recent manuscript explores the effects of EDs consumption on hemodynamic parameters [8]. The study also reports that as addiction increases, the hemodynamic effect of ED decreases. The negative effects of EDs on human health have been described and depend not only on the dose of caffeine but also on the presence of other energizing substances [7-9]. Energy drinks contain several ingredients, some known and declared on the label such as caffeine, glucose, taurine, B vitamins, and guarana (containing guaranine, similar to caffeine).

Interestingly, manufacturers are not required to include the caffeine content of some herbal supplements in their label because they are classified as “dietary supplements”. This involves partial information to the consumer about the content of exciting substances and leads to an increase in the amount of caffeine that is ingested. An example is guarana; both the plant and the berry have one of the highest levels of caffeine found in nature. Some manufacturers and companies use the word “guaranine” instead of caffeine, conveying correct but not entirely clear information for the consumers who have no specific knowledge. Since most EDs contain mixes of stimulants, mainly herbs that can interact with caffeine, more research is needed to determine the long-term effects of habitual ED intake, especially in young people. The further negative habit for human health is the association of ED with alcohol, thus predisposing many adolescents to alcohol addiction [10].

It has been speculated that caffeine intake may reduce alcohol-induced sedative effects and, therefore, drinkers may not experience symptoms of alcohol intoxication. This reduced perception seems to favor alcohol intake for longer times. Therefore, excessive EDs consumption associated with alcohol may expose you to greater likelihood of binge drinking. We agree with Sholeye *et al*. that “adequate caffeine control measures, with behavior change communication, will help to address this public health challenge among adolescents”.
